# Nanoparticle-mediated amelioration of drought stress in plants: a systematic review

**DOI:** 10.1007/s13205-023-03751-4

**Published:** 2023-09-08

**Authors:** Harsha K. Chandrashekar, Gunjan Singh, Arya Kaniyassery, Sachin Ashok Thorat, Roopa Nayak, Thokur Sreepathy Murali, Annamalai Muthusamy

**Affiliations:** 1 Department of Plant Sciences, Manipal School of Life Sciences, Manipal Academy of Higher Education (MAHE), Manipal, 576104 Karnataka India; 2 Manipal School of Life Sciences, Manipal Academy of Higher Education (MAHE), Manipal, 576104 Karnataka India; 3 Department of Biotechnology, Manipal School of Life Sciences, Manipal Academy of Higher Education (MAHE), Manipal, 576104 Karnataka India; 4 Department of Public Health Genomics, Manipal School of Life Sciences, Manipal Academy of Higher Education (MAHE), Manipal, 576104 Karnataka India

**Keywords:** Biochemical responses, Drought, Nanoparticle, NP uptake, Physiological traits, Osmoprotectants

## Abstract

Drought stress remains one of the most detrimental environmental constraints that hampers plant growth and development resulting in reduced yield and leading to economic losses. Studies have highlighted the beneficial role of carbon-based nanomaterials (NMs) such as multiwalled carbon nanotubes (MWNTs), single-walled carbon nanotubes (SWNTs), graphene, fullerene, and metal-based nanoparticles (NPs) (Ag, Au, Cu, Fe_2_O_3_, TiO_2_, and ZnO) in plants under unfavorable conditions such as drought. NPs help plants cope with drought by improving plant growth indices and enhancing biomass. It improves water and nutrient uptake and utilization. It helps retain water by altering the cell walls and regulating stomatal closure. The photosynthetic parameters in NP-treated plants reportedly improved with the increase in pigment content and rate of photosynthesis. Due to NP exposure, the activation of enzymatic and nonenzymatic antioxidants has reportedly improved. These antioxidants play a significant role in the defense system against stress. Studies have reported the accumulation of osmolytes and secondary metabolites. Osmolytes scavenge reactive oxygen species, which can cause oxidative stress in plants. Secondary metabolites are involved in the water retention process, thus improving plant coping strategies with stress. The deleterious effects of drought stress are alleviated by reducing malondialdehyde resulting from lipid peroxidation. Reactive oxygen species accumulation is also controlled with NP treatment. Furthermore, NPs have been reported to regulate the expression of drought-responsive genes and the biosynthesis of phytohormones such as abscisic acid, auxin, gibberellin, and cytokinin, which help plants defend against drought stress. This study reviewed 72 journal articles from 192 Google Scholar, ScienceDirect, and PubMed papers. In this review, we have discussed the impact of NP treatment on morphological, physio-biochemical, and molecular responses in monocot and dicot plants under drought conditions with an emphasis on NP uptake, transportation, and localization.

## Introduction

Food security remains a major concern for a country’s long-term and sustainable development. Sustainable agriculture is necessary to attain “Zero Hunger”, one of the United Nations’ 17 sustainable development goals. In the current global scenario, food production and distribution remain under severe strain because of the rising population, climate change, environmental contamination, and increased water and energy demands (Adrees et al. 2020; Usman et al. 2020; Van Nguyen et al. 2022). To add to this, current agricultural practices consume a large volume of resources. For example, although the annual crop production in the USA exceeds three billion tonnes, it requires 187 million tonnes of fertilizers, 4 million tonnes of pesticides, 2.7 trillion cubic metres of water (roughly 70% of all global freshwater), and over two quadrillion British thermal units (BTU) of energy (Kah and Hofman 2014). According to the FAO (2017), the world's population is expected to reach 10 billion by 2050, resulting in a 50% increase in food demand, particularly in developing nations. In developing countries, notably India, agriculture is one of the most essential components of the national economy. Increasing food production rates contribute significantly to the growth of the nation’s GDP. In addition, more than 60% of the population relies on it for sustenance, fodder, fuel, and fiber. The decline in food grain productivity can lead to food scarcity and a decline in nutrition security. Limitations in water and agricultural land availability are attributed as major reasons for declining food productivity trends, while the deterioration of water, soil nutrients, climate change, and so on can accentuate this problem (Bisht et al. 2022; Van Nguyen et al. 2022).

Nanotechnology could be a potential tool in remodeling various aspects of agriculture, from soil remediation to food packaging (Alabdallah et al. 2021). NPs can play various roles in agriculture and can be widely used as fertilizers, pesticides, herbicides, insecticides, growth regulators, nanocarriers, nanosensors, and nanobarcodes. Furthermore, nanotechnology can be applied in water filtration and soil remediation (Prasad et al. 2017; Al-Khayri et al. 2023). NPs can serve as cargo, and they can deliver genetic material and protein, resulting in genetic modification of medicinal and aromatic plants with higher resistance to stresses, as well as contributing to higher yield and enhanced nutrient uptake (Siddiqui et al. 2015; Al-Khayri et al. 2023). Furthermore, nanoscale materials can be used to monitor crop yield using geospatial techniques and nanosensors (Usman et al. 2020; Sharma et al. 2021). Nanobarcodes can tag proteins associated with pathogenicity, which can be used for rapid diagnostics and control of pathogen infections in crops (Hayat et al. 2023), making them key players in precision agriculture.

Sessile organisms such as plants are constantly exposed to an array of abiotic elements. Environmental variations such as drought, salinity, alkalinity, flooding, and mineral toxicity/deficiencies can cause stress to crops resulting in substantial yield reduction. Although some plants have the innate ability to withstand stresses, this is not the case with many plants (Hayat et al. 2023; Luz et al. 2023). Water is necessary for the plant life cycle as it is involved in nutrient transport. Stress caused by water deficit conditions due to physical lack of water, i.e., drought and physiological water inaccessibility, is most common in arid and semiarid regions (Luz et al. 2023). Drought stress impairs the photosynthesis, nutrient uptake, osmotic and antioxidant activities of plants. Photorespiration can lead to overproduction of reactive oxygen species (ROS) in drought-stressed plants leading to the denaturation of proteins, DNA damage, and lipid peroxidation, which hinders cell growth and elongation, resulting in poor plant growth and productivity (Waqas Mazhar et al. 2022; Hayat et al. 2023). Recent studies have highlighted the role of metal-based and carbon-based NPs in mitigating drought stress by inducing tolerance (Linh et al. 2020; Shekhawat et al. 2021). Carbon-based NMs such as graphene, fullerene, fullerol, and carbon NTs, and metal-based NPs, such as ZnO, TiO_2_, Fe, and Cu NPs, have been widely used to ameliorate drought stress by increasing water and nutrient uptake via stress tolerance and upregulation of genes involved in cell growth (Linh et al. 2020; Shekhawat et al. 2021).

NPs have been reported to enhance germination parameters, growth rate, biomass, and yield, regulate stomatal conductance, and transpiration rate and improve photosynthetic parameters. Furthermore, they reduce membrane ion leakage and enhance the assimilation of carbon dioxide in leaves (Aghdam et al. 2016; Borišev et al. 2016; Semida et al. 2021). In addition, NPs have been reported to regulate defense mechanisms by increasing the activities of enzymatic and nonenzymatic antioxidants such as catalase (CAT), superoxide dismutase (SOD), ascorbate peroxidase (APX), peroxidase (POD), and glutathione (GSH) (Taran et al. 2017; Djanaguiraman et al. 2018). Recent studies have shown that NPs ameliorate drought stress by decreasing the oxidative damage caused by the production of ROS (H_2_O_2_ and O_2_^−^) and additionally increasing the levels of osmolytes and osmoprotectants such as prolines, glycine betaine, soluble sugars, and amino acids that help in osmotic adjustment during drought stress conditions (Mustafa et al. 2021; Van Nguyen et al. 2022). The positive effects of carbon and metal-based NPs depend on their concentration, morphology, surface properties, mode of application, and type of plant species. In this review, we have compiled the current studies on NP-mediated drought mitigation and tolerance mechanisms to improve plant yield characteristics. Moreover, we have also highlighted the role of inorganic and organic nanoparticles in developing resilient crops for sustainable productivity.

## Materials and methods

**Fig. 1:**
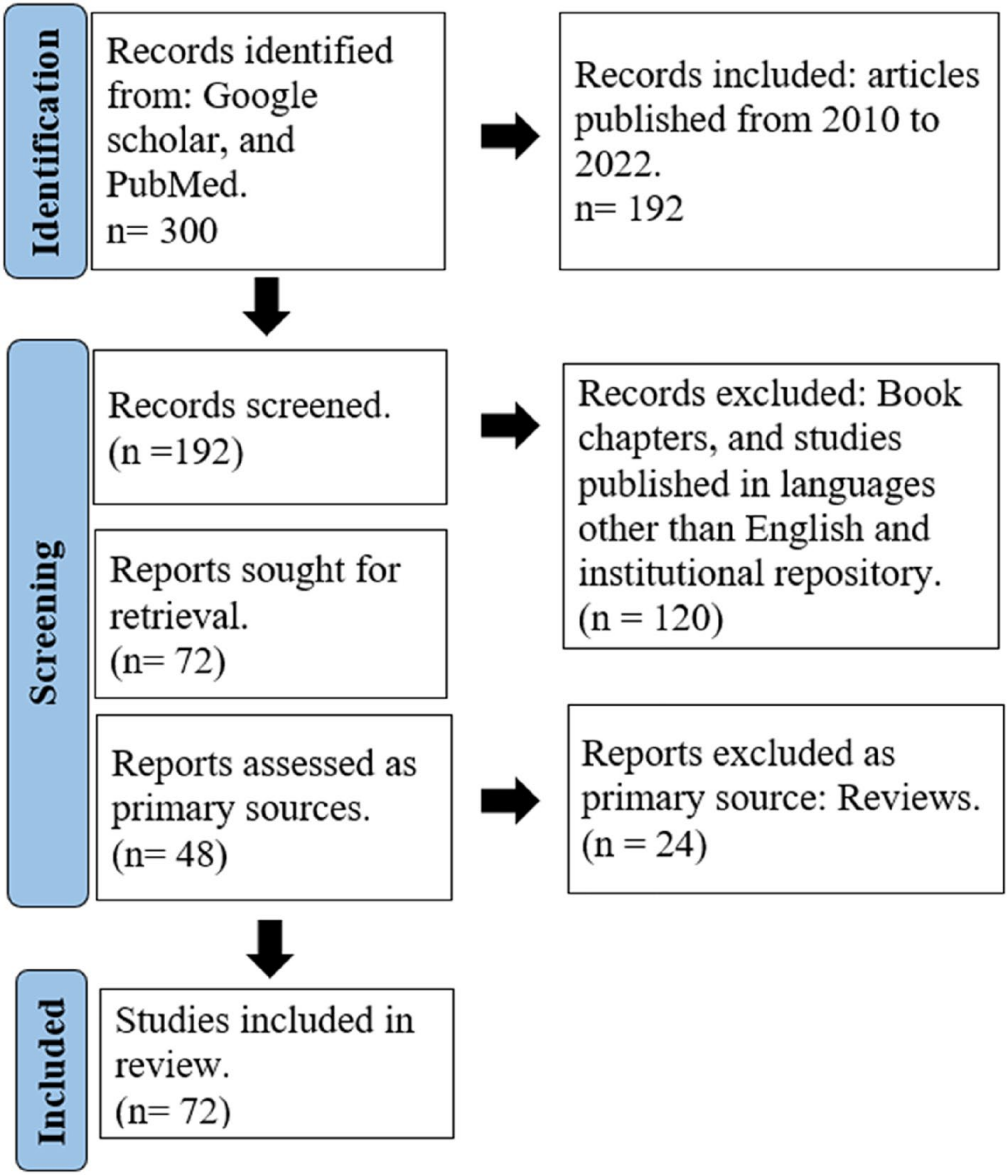
PRISMA diagram depicting the selection of studies for this review. The records were collected using the keywords, drought, nanoparticles, uptake and alleviation. The records were screened using the inclusion and exclusion criteria. The records used for this study as primary sources were strictly original articles, and the rest were used as secondary sources. (Adapted and modified from Page et al. 2021). Created using Microsoft word software

This literature review was carried out using major search engines, such as Google Scholar, Science Direct, and PubMed. The keywords inorganic nanoparticles, metal and metal oxide NPs, abiotic drought, seed priming, foliar treatment, uptake, lipid peroxidation, osmoprotectants and drought response gene were used for the search. Articles relevant to NP-mediated drought stress tolerance published in recent years (from 2010 to 2022) were only considered for our review. This resulted in 192 articles that were further screened, and studies in foreign languages, book chapters, conference proceedings, and institution repositories were excluded. After the screening, a total of 72 articles were considered, of which 48 reports were considered the primary source and 24 were reviews taken as secondary sources for this review (Fig. 1).

### Mechanism of uptake and transport of nanoparticles

Typically, the interactions between NPs and plants involve three stages: deposition, access, and translocation of NPs (Su et al. 2018). The adhesion of NPs onto plant surfaces is governed by van der Waals, hydrophobic, and electrostatic interactions. The uptake, translocation, and distribution of NPs primarily depend on several factors including the mode of application, concentration of nanomaterials, size, charge, and shape of the particle. Different modes of NP treatment for plants include seed treatment/priming, foliage treatment, soil treatment, irrigation, or hydroponics treatment (Fiol et al. 2021).

**Fig. 2:**
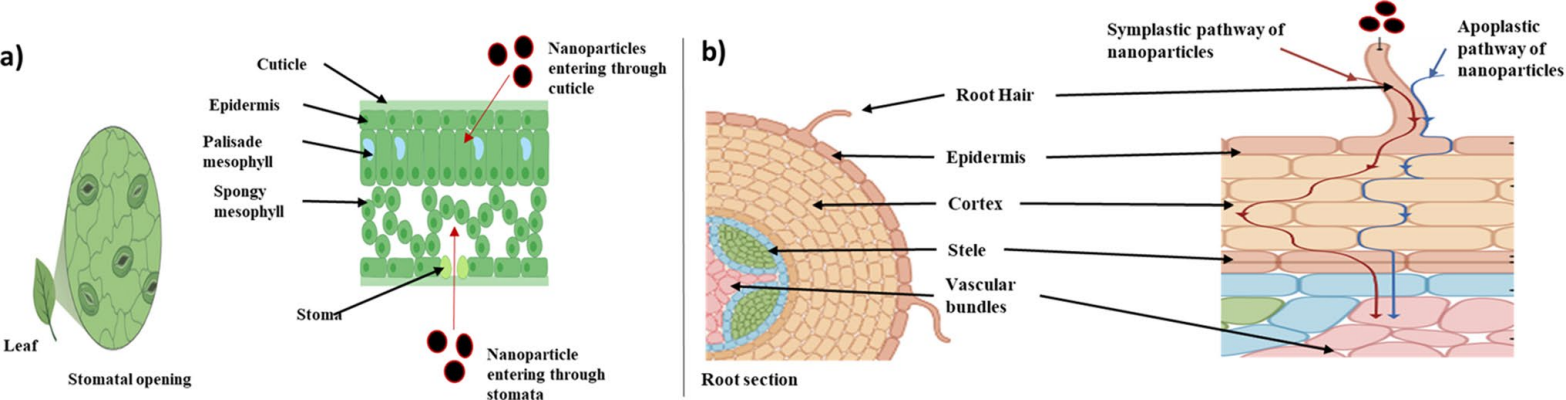
Uptake of nanoparticles in different parts of the plant, **a** Foliar-sprayed nanoparticles are taken up by plant cells by endocytosis either through stomata or cuticles present on the leaf surface. The nanoparticles can enter through stomatal opening or cuticle. **b** Two different pathways of nanoparticles in root system through symplastic and apoplastic pathways. (Adapted and modified from Usman et al. 2020). Created using BioRender.com and Microsoft PowerPoint software

Plant cells take up foliar-sprayed NPs by endocytosis through stomata or cuticles on the leaf surface. Stomatal pores are in the micrometer range, allowing uptake of larger NPs, whereas NPs below 5 nm size enter through a cuticular pathway (Avellan et al. 2019). The uptake of NPs through the foliar route also depends on leaf morphology, pore size, and stomatal density. Furthermore, the penetration of NPs into the cuticle and leaf mesophyll tissue is also influenced by their hydrophobicity, size, and chemical coating (Fig. 2a). For instance, PVP-coated gold (Au) NPs (3 nm) showed a higher cuticular penetration rate than citrate-coated Au NPs (Avellan et al. 2019). Variation in the rate of stomatal uptake in *Allium porum* leaves treated with polystyrene (43 nm) NPs was observed due to differences in stomatal abundance in various parts of the leaf (Avellan et al. 2019). Carbon-based NMs were taken up by plant cells through the pores present on the cell wall as the pore diameter was in the nano range (Ma et al. 2010). The internalization of TiO_2_ NPs (< 2.8 nm) through stomata was confirmed by X-ray fluorescence microscopy in in vitro cultivated *Arabidopsis thaliana* seedlings (Kurepa et al. 2010). The formation and deposition of nanoaggregates on the leaf surface were also observed, affecting the penetration rate. For example, entrapment of cerium oxide (CeO_2_) NPs was observed in cucumber leaf epidermis, leading to a reduction in the penetration rate up to < 30% (Hong et al. 2014). Furthermore, the translocation of NPs from leaf tissue to other plant parts occurs through vascular bundles (Hasaneen et al. 2016). Wang et al. (2013) reported stomatal uptake and translocation of metal oxide NPs (ZnO, Fe_2_O_3_) with a size range of 27.3–46.7 nm into stems and roots in watermelon plants following foliar exposure.

Following seedling and soil treatment, roots take up NPs through the apoplastic or symplastic pathway, osmotic pressure, capillary forces, or via the plant cell wall, depending upon the NP size (Fiol et al. 2021). In the apoplastic pathway, NPs enter plants through the root epidermis by penetrating the cell wall and cell membrane. Furthermore, the NPs entered the cortex, endodermis, and vascular bundles (Fig. 2b). However, casparian strips hinder NP penetration into the xylem, which can be bypassed through the symplastic route (Fiol et al. 2021). In the symplastic pathway, NPs enter root cells through endocytosis, passive diffusion, and facilitated transport mediated by aquaporins and ion channels. Furthermore, intercellular transport in the symplastic route occurs through the plasmodesmata.

NPs are transported within cells via endosomes. It has been hypothesized that this intracellular movement of NPs is regulated and directed toward the plasmodesmata by Rab proteins (Cifuentes et al. 2008). The transport of carbon, Au, and silver (Ag) NPs through the plasmodesmata route has been investigated in rice, poplar, and *Arabidopsis*, respectively (Geisler-Lee et al. 2013; Zhai et al. 2014). Li et al. (2016) reported the passive transport of Au NPs (< 20 nm) into root cells via cell wall pores with a diameter of 5–20 nm. Similarly, penetration of MWCNTs directly into cell membranes has been reported in *Catharanthus roseus* (Serag et al. 2012). The entry of NPs into root cells can also be mediated by endocytosis through clathrin-dependent pathways, (NPs ≤ 80 nm), and clathrin-independent pathways, (1000 nm). Alternatively, several metal ion transporters, including Nramps (natural resistance-associated macrophage proteins), IRT1 (iron regulated transporter 1), and COPT1 (copper influx transporter 1) that facilitate the transport of metal ions such as Fe^2+^/Fe^3+^, Zn^2+^, Mn^2+^, Cd^2+^, and Cu^+^/Cu^2+^ have been identified in plants suggesting their involvement in the transport of metal-based NPs (Wang et al. 2012). Following uptake, NPs are translocated from roots to aerial parts of the plant via xylem and phloem sieve tube elements along with water and sap (Ali et al. 2021). Wang et al. (2012) reported unidirectional and bidirectional transport of CuO NPs via xylem and phloem in *Zea mays*.

Similarly, the transmission of MWCNTs from roots to stems and leaves via the vascular system was reported in *Onobrychis* seedlings (Smirnova et al. 2011). Fluorescence microscopy and FT-IR (Fourier transform infrared) studies have been widely employed to study the uptake and translocation of carbon NMs (Serag et al. 2012). Sap composition, ionic strength, and flow rate influence NP dissolution, aggregation, and transformation within the phloem (Su et al. 2018). For instance, the presence of inorganic substances (PO_4_^3−^, S^2−^) in the sap can lead to the transformation of iron NPs. Similarly, phloem sap containing organic acids inhibits the disintegration of Ag NPs (de la Rosa et al. 2021). NP uptake and transport are also species dependent. For example, the deposition of Au NPs was observed in shoots of *Oryza sativa* but not in *Cucurbita pepo* (Zhu et al. 2012). Similarly, the transport of CeO_2_ NPs was observed in pumpkin but not in wheat (Schwabe et al. 2013). Several reports suggest that this variability in NP transport among different plant species might be due to differences in size exclusion limit (SEL) and morphological characteristics (Fiol et al. 2021). However, the exact mechanism behind interspecies variation in NP uptake and transport is still under investigation.

### Impact of nanoparticles on plants under drought stress

**Fig. 3:**
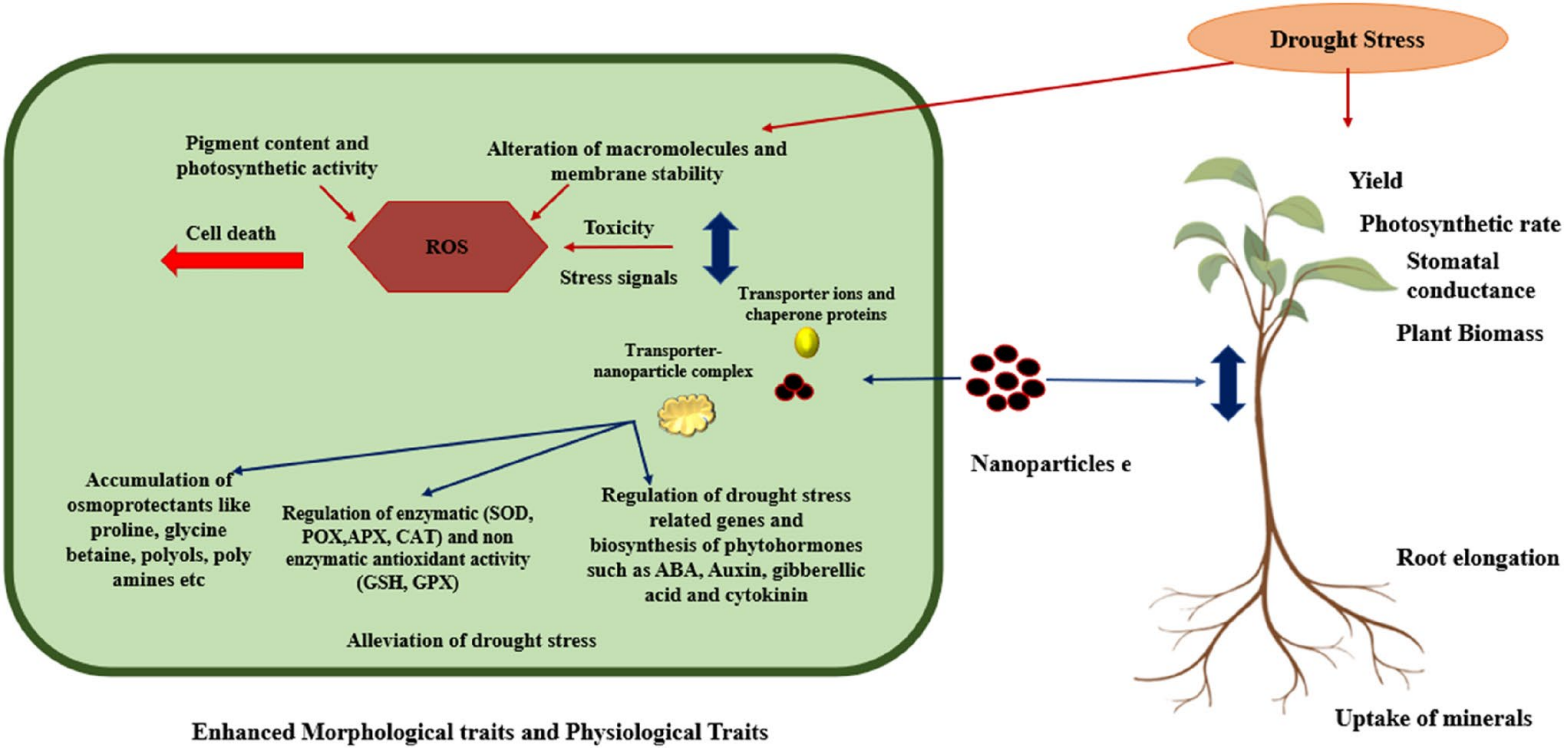
Impact of nanoparticles on plant and cells under drought stress including signalling pathways such as regulation of defense mechanism, antioxidant activity and drought responsive genes and biosynthesis of phytohormones. Drought exposure triggers the accumulation of Reactive Oxygen Species (ROS) which causes oxidative stress. ROS alters the macromolecules in the cytoplasm and degrades the cell membrane. In addition, it decreases the pigment content thus reducing the photosynthetic activity. Prolonged oxidative stress ultimately leads to cell death. Overall effects of drought can be seen on the plant including reduced yield, wilting, stunted growth and plant death. The nanoparticles upon treatment, enter the cell and form a complex with transporter ions and regulate the accumulation of osmoprotectants, increase the activity of antioxidants, biosynthesis of hormones and regulation of drought responsive genes. (Adopted and Modified from Jalil and Ansari 2019). Created using BioRender.com and Microsoft PowerPoint software

Water plays a vital role throughout a plant's life, from seed germination to the flowering/fruiting stage. Plant water deficit occurs when water loss occurs through the transpiration process or due to an impaired root system. Water deficit can impair morphological, physiological, and biochemical traits and molecular processes in plants (Seleiman et al. 2021; Hayat et al. 2023). Typically, plants under drought stress exhibit discoloration of leaves, leaf rolling, stunted growth, and permanent wilting. Recent studies have shown that the application of nanoparticles alleviates drought stress and enhances agronomical, physiological, and biochemical mechanisms (Jalil and Ansari 2019) (Fig. 3). The efficiency of NPs depends on their concentration, chemical composition, size, and morphology (Siddiqi et al. 2015). The larger surface area and smaller size make the NPs highly reactive when compared to their bulkier counterparts (Usman et al. 2020). All the recent studies where NPs have alleviated drought stress by regulating the germination, morphology, physio-biochemical, and molecular parameters have been compiled in Tables 1 and 2.

**Table 1:** Impact of nanoparticles on germination, morphological physio-biochemical and molecular parameters of dicot plants under drought stress

Nanoparticles (NPs)	Concentration of nanoparticles (NPs)	Name of crop/family	Mode of treatment	Response/tolerance mechanism	References
Nano Zinc oxide (ZnO NPs)	0, 0.5, and 1 g/L (ZnO NPs)	Soybean (*Glycine max*, Fabaceae)	Seed treatment	Enhanced germination percentage and rate	Sedghi et al. (2013)
Nano TiO_2_ (nano-anatase)	0, 10, 100, and 500 mg/L	Flaxseed or Linseed (*Linum usitatissimum* L., Linaceae)	Soil treatment	Increased photosynthetic parameters and pigments. Decreased ROS (reactive oxygen species) and increased the accumulation of MDA (Malondialdehyde)	Aghdam et al. (2016)
Silica Nanoparticles (SiO_2_ NPs)	0, 10, 50, and 100 mg/L	Hawthorn (*Crataegus laevigata*, Rosaceae)	Soil treatment	Improves photosynthetic rate and stomatal conductance. Decreased MDA and xylem water potential	Ashkavand et al. (2015)
Fullerenol nanoparticles -FNPs	700 μmol/L and 70 μmol/L	Sugar beet (*Beta vulgaris,* Amaranthaceae)	Foliar treatment	Accumulation of GSH (Glutathione) and MDA Decreased antioxidant activities of CAT, APx and GPx	Borišev et al. (2016)
Micnobits (ZnO, CuO, and B_2_O_3_ NPs)	1.77, 0.80, and 0.92 g/L	Soybean (*Glycine max,* Fabaceae)	Foliar treatment	Enhanced uptake of NPK as well as S, Ca, and Mg	Dimpka et al. (2020)
Maghemite particles Y-Fe_2_O_3_ NPs	0.5, 0.8, 1, or 2 mg/mL, totally 100, 160, 200 and 400 mg per plant	Rapeseed (*Brassica napus*, Brassicaceae)	Hydroponics treatment	Reduced harmful Fenton reactions (Iron salts and hydrogen peroxide in acid conditions) Scavenged reactive oxygen species	Palmqvist et al. (2017)
TiO_2_ NPs	20 ppm	Mullein (*Verbascum sinuatum*, Scrophulariaceae)	Hydroponics treatment	Increased flavonoids and total phenolic contents. Enhanced photosynthetic pigments. Improved nitrogen assimilation	Karamian et al. (2019)
Fe, Cu, Co, ZnO Nanoparticles	50 mg/L of Fe, ZnO and Cu NPs and 0.05 mg/L, Co NPs, ZnO NPs	Soybean (*Glycine max*, Fabaceae)	Seed treatment	Enhanced physiological traits such as, relative water content, drought tolerance index, and biomass reduction rate, Upregulated drought tolerance marker genes, GmRD20A, GmDREB2, GmERD1, GmFDL19, GmNAC11, GmWRKY27, GmMYB118, and GmMYB174 in roots or shoots (or both). Upregulated ABA biosynthesis	Linh et al. (2020)
ZnO NPs	0, 50, and 100 ppm	Eggplant (*Solanum melongea* L. Solanceae)	Foliar treatment	Improved acquisition of macro-and micronutrients, increasing relative water content (RWC), alleviating cell membrane damage Increased in biomass. (Leaf, stem etc.)	Semida et al. (2021)
Silicon nanoparticles	0, 100, 200, and 500 mg/L	Marigold (*Calendula officinalis,* Astraceae)	Seed treatment	Enhanced germination rate and germination index. The vigour index based on seedlings length and dry weight	Rahimi et al. (2021)
Selenium nanoparticles	20 mg/L	Pomegranate (*Punica granatum*, Lythraceae)	Foliar treatment	Enhanced photosynthetic parameters. Increased phenolic content, antioxidants, osmolytes and ABA levels	Zahedi et al. (2021)
Silicon nanoparticles	1.5 mM	Coriander (*Coriandrum sativum L*. Apiaceae)	Foliar treatment	Improved plant growth and yield. Enhanced relative water content, total soluble sugar, total phenolic content, total flavonoid content, essential oil content	Afshari et al. (2021)
Chitosan nanoparticles	1%	Periwinkle (*Catharanthus roseus*, Apocyanaceae)	Foliar treatment	Enhanced plant growth, increased total chlorophyll content and increased photosynthetic rateIncreased plant growth, relative water content, stomatal conductance, and total chlorophyll. Increased proline accumulation and antioxidant activity of CAT and APX. Reduced H_2_O_2_ and MDA accumulation. High alkaloid content was associated with induced gene expression of strictosidine synthase (STR), deacetylvindoline-4-*O*-acetyltransferase 15(DAT), peroxidase 1 (PRX1) and geissoschizine synthase (GS)	Ali et al. (2021)
Zinc oxide nanoparticles	25, 50, and 100 mg/L	Tomato (*Solanum lycopersicum* L, Solanaceae)	Foliar treatment	Increased biomass. Reduced malondialdehyde and hydrogen peroxide content. Increased ascorbic acid, free phenols, and the activity of SOD, CAT, and APX	El-Zohri et al. (2021)
Natural char nanoparticles	0.3 and 0.6%	Tomato (*Solanum lycopersicum* L, Solanaceae)	Soil treatment	Increased plant growth, nutritional indices, increased soil microbial population	Nassaj-Bokharaei et al. (2021)
Magnetite nanoparticles	20, 50, 100, and 200 mg/L	Fenugreek (*Trigonella foenum-graecum*, Fabaceae)	Foliar treatment	Enhanced plant growth, increased total chlorophyll content and increased photosynthetic rate	Bisht et al. (2022)
Zinc oxide nanoparticles	25 mg/L and 100 mg/L	Cucumber (*Cucumis pepo,* Cucurbitaceae)	Foliar treatment	Decrease in ROS and Peroxidation. Increased glycine betaine, proline, total amino acids, and soluble sugars	Ghani et al. (2022)
Silicon dioxide nanoparticles	12.5, 25, and 50 ppm	Green Pea (*Pisum sativum,* Fabaceae)	Foliar treatment	Improved growth parameters. Increased RWC, specific leaf area. Increased activity of CAT, SOD, APX and GSH. Increased phenolic contents. Reduced hydrogen peroxide and lipid peroxidation	Sutulienė et al. (2022)
Selenium nanoparticles	0.5, 1.5, 3, 4.5, and 6 mg/L	Quinoa (*Chenopodium quinoa*, Chenopodiacea)	Seed treatment	Increased in germination parameters and antioxidant enzyme activity (Catalase (CAT), superoxide dismutase (SOD), and ascorbate peroxidase (APX)), proline, and protein content	Gholami et al. (2022)
Zinc oxide nanoparticles	10, 20, 40, 50, 100, 300, and 1000 ppm	Peanut (*Arachis hypogea*, Fabaceae)	Foliar treatment	Increased biomass and pod yield and promoted antioxidant enzyme activity	Latha et al. (2022)

**Table 2:** Impact of nanoparticles on germination, morphological physio-biochemical and molecular parameters of monocot plants under drought stress

Nanoparticles (NPs)	Concentration of NPs	Name of crop /family	Mode of treatment	Response/tolerance mechanism	References
Cu NPs and Zn NPs	1%	Winter wheat (*Triticum aestivum*, Poaceae)	Seed treatment	Decreased Thiobarbituric acid reactive substances (TBARS). Increased antioxidative enzyme (SOD and catalase) activity. Increased carotenoids	Taran et al. (2017)
Nanoceria- Cerium oxide nanoparticles	10 mg/L	Sorghum (*Sorghum bicolor*, Poaceae)	Foliar Treatment	Decreased ROS, hydrogen peroxide and MDA. Increased the activities of CAT (Catalase), SOD (superoxide dismutase) and POD (peroxidase). Decreased photosynthetic rates and stomatal conductance	Djanaguiraman et al. (2018)
Chitosan nanoparticles containing GSNO	100 µM	Sugarcane (*Saccharum officinarum*, Poaceae)	Hydroponics treatment	Increased Leaf CO_2_ assimilation. Enhanced stomatal conductance and increased relative water content	Silveira et al. (2019)
ZnO NPs	2.17 mg/kg	Wheat (*Triticum aestivum*, Poaceae)	Soil treatment	Increased uptake of Zn, N and P	Dimpka et al*.* (2020)
TiO_2_ NPs	500, 1000, and 2000 mg/kg	Wheat (*Triticum aestivum*, Poaceae)	Soil treatment	Increased seedling length (SL), superoxide dismutase (SOD) activity, total soluble proteins, net photosynthetic rate and intercellular CO_2_ concentration (Ci)	Faraji and Sepehri (2020)
Selenium nanoparticles	10, 20, 30, and 40 mg/L	Wheat (*Triticum aestivum,* Poaceae)	Foliar treatment	Enhanced morphological traits such as root/shoot length, leaf number and area, fresh/dry weight of root and shoot	Ikram et al. (2020)
Silver and copper nanoparticles	0, 3, 5 and 7 mg/L Cu NPS and 0, 10, 20 and 30 mg/L Ag NPS	Wheat (*Triticum aestivum*, Poaceae)	Hydroponic treatment	Enhanced morphological traits, stomatal conductance and chlorophyll stability index, leaf succulence and leaf K content	Ahmed et al. (2021a, b)
Iron oxide nanoparticles	0, 25, 50, and 100 mg/kg	Rice (*Oryza sativa,* Poaceae)	Soil treatment	Increased biomass, photosynthetic efficiency, antioxidant enzymes, uptake of nutrients. Decreased ROS	Ahmed et al. (2021a, b)
Titanium oxide nanoparticles	20 and 40 ppm	Wheat (*Triticum aestivum*, Poaceae)	Soil treatment	Increased biomass, chlorophyll content, RWC, MSI, antioxidant enzymes, and osmolyte content	Mustafa et al. (2021)
Zinc oxide nanoparticles + biochar	100 mg/L	Wheat (*Triticum aestivum*, Poaceae)	Soil treatment	Improved wheat growth and biomass, chlorophylls contents, antioxidant enzyme and reduced activities by scavenging ROS	Bashir et al. (2021)
Zinc oxide nanoparticles	100 mg/L	Maize *(Zea mays,* Poaceae)	Soil treatment	Reduced photosynthetic pigment degradation and regulated the stomatal movement, maintained a higher net photosynthetic rate, and enhanced water use efficiency. Enhanced activities of UDP-glucose pyrophosphorylase, phosphoglucoisomerase and cytoplasmic invertase which enhanced the biosynthesis of starch and sucrose and regulated glycolysis	Sun et al. (2021)
Graphene oxide nanosheet + PGPB	0.52 mg/g	Maize (*Zea mays*, Poaceae)	Soil treatment	Increased shoot biomass. Increased proline, activities of SOD and CAT. Increased ABA. Enhanced photosynthetic parameters	Lopes et al. (2021)
Selenium nanoparticles	100 µg/mL	Wheat (*Triticum aestivum*, Poaceae)	Soil treatment	Enhanced plant growth, grain quantity and quality. photosynthetic pigments and gas exchange parameters	El-Saadony et al. (2021)
Silicon nanoparticles + PGPR	150 mg/kg	Wheat (*Triticum aestivum*, Poaceae)	Seed and soil treatment	Improved biomass, & chlorophyll-a, & b. Improved relative water content, gas exchange attributes, nutrients uptake, and osmolytes production. Increased the antioxidant enzymes activities such as, SOD, POD, & CAT	Akhtar et al. (2021)
Zinc oxide nanoparticles and bulk zinc sulphate	15 mg/L	Wheat (*Triticum aestivum*, Poaceae)	Seed treatment	Prevented chlorophyll degradation, improved photosynthetic parameters and plant growth. Decreased the activity of antioxidant enzymes- CAT), POD, SOD, & GR as well as MDA content. Upregulated H_2_O_2_ signalling pathway	Rai-Kalal and Jajoo (2021)
Copper nanoparticles	52, 69.4, and 86.8 µM	Maize (*Zea mays*, Poaceae)	Seed treatment	Increased leaf water content and plant biomass. Increased anthocyanin, chlorophyll and carotenoid content and reduced ROS	Van Nguyen et al. (2022)
Iron oxide nanoparticles	0.3, 0.6, 0.9, and 1.2 mM	Wheat (*Triticum aestivum*, Poaceae)	Seed treatment	Increased in chlorophyll a and b, carotene. Increased in free proline, antioxidant levels (SOD and APX) and total protein content. Decreased in lipid peroxidation and electron leakage	Noor et al. (2022)
Zinc oxide nanoparticles	10 ppm	Wheat (*Triticum aestivum*, Poaceae)	Seed treatment	Increased biomass, photosynthetic pigments, nutrients, soluble sugars, proteins, ABA and indole acetic acid content. Increased proline, antioxidant enzymes (SOD, CAT, APX, GSH & DHAR). Reduced electrolyte leakage, MDA and H_2_O_2_	Azmat et al. (2022)
Iron nanoparticles	5, 10, and 15 mg/L	Wheat (*Triticum aestivum*, Poaceae)	Seed treatment	Increased rhizosphere colonization level, water use efficiency and photosynthetic rate. Increased biomass	Naseer et al. (2022)
Silicon dioxide nanoparticles	150 mg/L	Wheat (*Triticum aestivum*, Poaceae)	Seed treatment	Increased germination percentage, germination index, and germination vigour index. shoot length and root length. Increased photosynthetic pigments, osmolytes content, relative water content, membrane stability index, phenol, flavonoid content, indole acetic acid and cytokinin. Increased in activity of CAT, POD, & SOD	Akhtar and Ilyas (2022)
Titanium dioxide and zinc oxide nanoparticles	5 and 10 mg/L	Wheat (*Triticum aestivum*, Poaceae)	Seed treatment	Increased morphological parameters, grain yield, and crop water productivity. Increased in photosynthetic pigments. Increased in IAA content, proline, total soluble sugars and amino acids. Regulation of protein synthesis	El-Bassiouny et al. (2022)
Zinc oxide nanoparticles	5, 10, 15, 25, and 50 ppm	Rice (*Oryza sativa,* Poaceae)	Seed treatment	Increase in plant height, total chlorophyll contents, plant fresh and dry weights. Increased panicle length, number of tillers, paddy yield and straw yield. Reduced MDA. Increased proline content, activities of SOD, CAT, & POD	Waqas Mazhar et al. (2022)

### The effects of nanoparticles on germination and vegetative traits under drought stress

Seed germination, the crucial stage for seedling establishment and the first stage of a plant's life is highly sensitive to environmental stresses. Drought stress causes delayed germination due to decreased water uptake in seeds, leading to the inactivation of hydrolytic enzymes, particularly amylase which is necessary for embryo development (Sedghi et al. 2013; Rahimi et al. 2021; Gholami et al. 2022). NPs have been reported to positively affect crop plant germination and growth rates (Sedghi et al. 2013). The precise mechanism behind this is yet to be investigated. In this review, recent reports on the NP-mediated enhancement of germination and growth parameters of crop plants under drought stress have been compiled and critically analyzed. Seeds primed with NPs have been reported to show higher germination rates, probably due to the enhanced production of phytohormones acting as germination promoters (Gholami et al. 2022). ZnO NP-treated soybean exhibited an enhanced germination rate compared to the control groups. ZnO NP treatment (1 g/L) increased the germination percentage and rate by 89.5% in plants under severe PEG-induced drought stress of − 1 MPa. It was reported that Zn^2+^ acts as a cofactor in activating hydrolytic enzymes, resulting in an improved rate of embryonic development. The radicle length was reportedly high in 1 g/L treated soybean seedlings (Sedghi et al. 2013). In addition, the study reported a significant increase in the fresh and dry weight of soybean seedlings with increasing ZnO NP concentration. Moreover, Sedghi et al. (2013) also suggested using ZnO NPs for enhanced membrane stability and increased cell elongation. The residual weight of fresh and dry samples decreased with increasing nanoparticle concentration, suggesting that seed reservoirs in ZnO NP-treated seeds were consumed efficiently to biosynthesize phytohormones (auxin and gibberellin) in the presence of Zn^+2^ ions. Subsequently, an increased growth rate of plumule and radicle length in the soybean seedlings was observed. It has been hypothesized that NPs activate the amino acid tryptophan, which plays a crucial role in the biosynthetic pathways of auxin, leading to cell division during seedling growth (Waqas Mazhar et al. 2022).

Morphological traits such as growth rate, radicle length, seed vigor index, fresh weight, dry weight, seedling growth, and mean germination time were enhanced upon treatment with a low concentration of carbon nanotubes (CNTs) in *Alnus subcordata*, hopbush, chickpea, and wheat (Tripathi et al. 2011; Rahimi et al. 2016; Yousefi et al. 2017; Joshi et al. 2018). Wenli et al. (2020) reported that treating soybean seeds with SWCNTs under low water potential could increase the germination index and root and shoot lengths. Ali et al. (2020) highlighted the possibility of CNTs to remodel membrane lipids and seed membranes which could contribute to an improved germination rate. The nanopriming of Labrador tea (*Rhododendron groenlandicum* L.) and Bog birch (*Betula pumila* L.) seeds with MWCNTs alleviated seed dormancy and improved the germination rate and seed vigor. On assessing the lipidome of the treated and control seedlings, there was an increase in plastidic lipids, such as phosphatidylcholine (PC), phosphatidylglycerol (PG), and lysophosphatidylcholine (LPC) (Ali et al. 2020).

Marigold seeds treated with bulk silica and silica NPs (SiO_2_) showed a positive effect on germination parameters, such as germination percentage, rate, vigor index of seedling, weight, and length. The germination rate was higher in seeds treated with SiO_2_ NPs than in bulk Si particle-treated seeds. The rest of the germination parameters did not show a noticeable difference between bulk SiO_2_ and SiO_2_ NP-treated seeds. However, there was significant enhancement compared to the control group under severe drought stress of 1.5 MPa. The higher germination rate observed in SiO_2_ NP-treated seeds suggests that the NPs could improve the water uptake by regulating aquaporins on the seed coat. Both SiO_2_ and SiO_2_ NPs were shown to be involved in the biosynthesis of phytohormones, such as auxins and gibberellins, resulting in the enhanced germination percentage and vigor index of marigold seedlings in both treatments. In contrast, reduced germination parameters were observed in the control group under severe drought conditions (Rahimi et al. 2021).

Faraji and Sepehri (2020) reported the enhancement in biomass of wheat seedlings treated with different concentrations of TiO_2_ nanoparticles under moderate and severe drought stress along with sodium nitroprusside ((Na_2_[Fe(CN)_5_(NO)])) as a nitrous oxide (NO) donor. Under moderate stress, NP-treated seedlings showed a significant increase in the length of wheat seedlings; however, this was not the case under severe stress. The seedlings treated with NPs and sodium nitroprusside under severe drought stress showed a higher seedling length than seedlings treated with only NPs. However, the control group showed decreased seedling length compared to the seedlings treated with NP alone and NP along with sodium nitroprusside. Sodium nitroprusside was suggested to provide NO and aid in the increased growth of wheat seedlings along with TiO_2_ NP treatment, thus improving the protective effect of TiO_2_ NPs. The foliar application of biosynthesized nano selenium (Se) to wheat seedlings improved various morphological traits, such as root and shoot length, plant height, total number of leaves, leaf area, and fresh and dry weight of seedlings under water deficit conditions. Treatment with Se NPs at a concentration of 30 mg/L Se NP enhanced the morphological traits of wheat seedlings under water deficit conditions compared to other concentrations. The biosynthesized Se NPs at a lower dose promoted root‒shoot growth, while organogenesis was stimulated by the accumulation of nano selenium in the seedlings (Ikram et al. 2020). The agronomic traits of Cu and Ag NP-treated wheat plants were shown to be enhanced compared to those of control group under drought stress. The lower concentration (3 mg/L) treatment with Cu NPs and a higher concentration (30 mg/L) treatment with Ag NPs improved the spike number and spike length (Ahmed et al. 2021a, b). Recent studies have shown the positive effect on germination, growth, and agronomical parameters in crop plants treated with metal/metal oxide particles. The enhancement of these parameters could be attributed to the dose-dependent uptake and accumulation of these metal/metal oxide NPs and their interaction with plant cells.

### The effects of nanoparticles on physio-biochemistry under drought stress

NP treatment has been reported to alleviate drought stress by enhancing physiological parameters, including improved water relations, stomatal conductance, photosynthetic activity, and uptake of micro- and macronutrients (Siddique et al. 2016). Carbon NMs, including graphene, were shown to significantly increase the chlorophyll content, thereby enhancing light absorption, flow of electrons, and photosynthetic activity in *Paeonia ostii* (Zhao et al. 2020). Linseed seedlings grown in soil treated with TiO_2_ NPs showed enhanced pigment content under both well-watered and water-deficit conditions. Chlorophyll a and b were notably lower in water-deficit seedlings, but the carotene content was higher in water-deficit plants. TiO_2_ NPs have been suggested to trigger the accumulation of carotene pigment in water-deficit plants. The literature suggests that TiO_2_ modifies the cell wall to increase fluidity, thereby aiding in cell expansion (Mustafa et al. 2021). Seed and shoot analysis showed that the NP-treatment reportedly enhanced the uptake of K and P under both well-watered and water-deficit conditions. TiO_2_ NPs have been suggested to enter the root system and increase soil nutrient uptake in linseed plants (Aghdam et al. 2016). The high surface reactivity of NPs is attributed to enhanced pores in the roots, resulting in increased inflow of water and nutrients and improving seedling growth and development despite stress (Mustafa et al. 2021). SiO_2_ NPs have been shown to increase the xylem potential in Hawthorne seedlings. Although there were no significant changes in the relative water content of water-stressed plants compared to controls, the negative effect of drought stress was shown to have reduced effect on Hawthorne seedlings due to the increased xylary potential (Ashkavand et al. 2015). Si NPs Si NP seed priming enhanced the length of wheat seedlings during the crucial stages of water deficit conditions. It has been reported that Si NPs accumulate on the cuticle, forming a barrier, protecting seedlings from stress and retaining water by decreasing transpiration. Si NPs were also shown to improve water and nutrient uptake which might have resulted in enhanced biomass in the seedlings (Raza et al. 2023). Furthermore, Si NPs have been reported to increase endogenous cytokinin, which aids in the restoration of photosynthetic pigments despite stressful conditions.

In another study, drought and oxidative stress were mitigated by foliar application of fullerenol NPs in *Beta vulgaris*. These nanoparticles bind to water molecules, forming an additional water reserve that helps plants cope with water stress (Borišev et al. 2016). *Salvia mirzayanii* seeds treated with graphene oxide/polyaniline (GO/PANI) nanocomposites improved physiological traits such as the membrane stability index under water deficit conditions (Hatami et al. 2019). It has been suggested that carbon NTs nullify the adverse effects of drought stress through their interaction with aquaporins by promoting water uptake (Martínez-Ballesta et al. 2016). Studies on *Hyoscyamus niger* have reported that oxidative injury induced by drought stress can also be overcome by seed priming with SWCNTs, as it can activate the plant antioxidant machinery by penetrating inside the seed coat. Furthermore, treatment with CNTs can increase the content of chlorophyll, protein, phenolic compounds, and proline which helps plants overcome and survive unfavorable conditions (Hatami et al. 2019). Augmentation of plant antioxidative status by improving ascorbic acid and glutathione content and reducing ROS and MDA content has been observed in fullerol-treated *Brassica napus* and *Zea mays* plants grown under water stress (Xiong et al. 2018). The relative water content, drought tolerance index, and biomass reduction rate were analyzed in soybean seedlings treated with Fe, Cu, Co, and ZnO NPs. All groups of seedlings showed similar relative water content; however, Fe and Cu-treated soybean seedlings showed a higher relative water content of 71%. With the accumulation of Cu and Fe NPs, the water retention capacity of seedlings was also enhanced.

Fe NP-treated soybean seedlings showed the highest drought tolerance index compared to Cu, Co, and ZnO NP-treated and control groups. The biomass reduction rate was significantly lower in Fe and Co NP-treated soybean seedlings under water deficit conditions. In addition, Fe NPs were found to be more efficient in alleviating drought stress in soybean than Cu, Co, and ZnO NPs (Linh et al. 2020). Foliar application of ZnO NPs on eggplant could improve photosynthetic activity in both well-watered and drought-stress conditions compared to the control groups. The plants in the negative control group showed a decline in the leaf chlorophyll index; however, ZnO NPs improved the chlorophyll index under water deficit conditions and improved the photosynthetic parameters. In addition, ZnO NPs have been reported to improve membrane stability under stress and play a significant role in maintaining the relative water content in seedlings compared to control groups. It was found that treatment with 100 ppm NPs could notably increase the physiological parameters in eggplant seedlings under drought stress (Semida et al. 2021). Sallam et al. (2019) and Ahmed et al. (2021a, b) reported that wheat seedlings showed enhanced photosynthetic activity with elevated chlorophyll a and b pigments when treated with Cu and Ag NPs. Previous studies have reported that Cu is a vital micronutrient that is involved in photosynthesis. Wheat seedlings treated with Cu NPs exhibited regulated stomatal conductance at 0.3 mg/L under drought stress as well as enhanced photosynthetic activity thus alleviating drought stress (Ahmed et al. 2021a, b).

Abiotic stress, including drought, can cause oxidative damage by producing O_2_, OH^−^ and H_2_O_2_ radicals. The ROS produced can severely injure plant tissues and can damage carbohydrates, lipids, and proteins, leading to decreased metabolism and membrane damage. ROS can cause irrecoverable damage by injuring DNA and triggering programmed cell death in plant tissues (Khaleghi et al. 2019). An increase in the accumulation of H_2_O_2_ causes lipid peroxidation, which in turn causes membrane damage (Ashkavand et al. 2015) that can be measured by the accumulation of MDA. TiO_2_ NP-treated linseed seedlings inhibited the accumulation of H_2_O_2_ under both well-watered and drought conditions compared to the control groups. Cellular electron exchange mechanisms were enhanced with the decrease in ROS generation, and accumulation of MDA. TiO_2_ NPs at a concentration of 4 mM reduced oxidative damage caused by ROS production and electron leakage (Aghdam et al. 2016). Hawthorne seedlings treated with SiO_2_ NPs exhibited reduced MDA accumulation in under-watered and stressed conditions. The MDA content in the seedlings declined with increasing concentrations of SiO_2_ NPs.

**Fig. 4:**
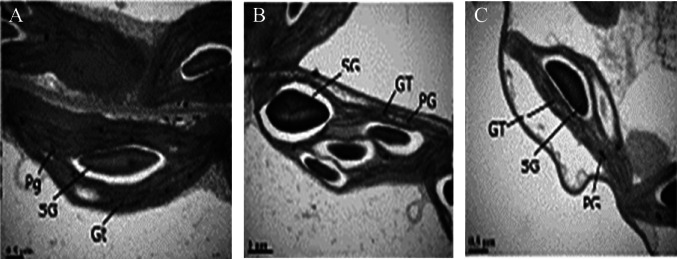
**A** Ultra structure of the chloroplast (control) observed under TEM. The appearance of the chloroplast is oblong shape which is normal. The structural integrity of the chloroplast is intact. **B** Ultra structure of the chloroplast (drought stressed). The chloroplast appears to be irregular with fewer number of grana. The thylakoids appear to be swollen. **C** Ultra structure of the chloroplast (Ca NP treated). The structural integrity appears to be better and more regular. The thylakoids don’t appear to be swollen (Adapted from Ayyaz et al. 2022, Reproduced with the permission from publisher)

SiO_2_ NPs are reported to enhance the antioxidant activities of catalase, peroxidase, and superoxide dismutase. By decreasing electrolyte leakage in Hawthorne seedlings, SiO_2_ NPs protected the seedlings from oxidative damage caused by drought stress (Ashkavand et al. 2015). Under drought stress, rapeseed seedlings treated with maghemite (iron oxide) NPs (Fe_2_O_3_) exhibited reduced accumulation of H_2_O_2_ and MDA compared to the control. This reduced the lipid peroxidation caused by the production and accumulation of H_2_O_2_ (Palmqvist et al. 2017). The rapeseed seedlings treated with Ca NPs under 15% PEG induced drought stress improved photosynthetic parameters such as pigment contents, photosynthetic rate and photosystem performance. The seedlings were observed under transmission emission microscope (TEM), to analyse the effects of Ca NPs on the cell organelles. The chloroplast under drought appeared to be irregular with swollen thylakoids as chloroplast is more susceptible for oxidative damages (Fig. 4). The Ca NP-treated seedlings showed the chloroplast was more regular compared to stressed plant cell (Ayyaz et al. 2022). Wenli et al. (2020) reported that the treatment of soybean seeds with SWCNTs increased SOD, CAT, and POD activity which ultimately helped alleviate drought stress. Foliar treatment of ZnO NPs in cucumber reduced the accumulation of H_2_O_2_ and (O_2_)^−^ under drought stress conditions. Treatment with 100 mg/L ZnO NPs reportedly exhibited a 36% reduction in H_2_O_2_ and a 40% reduction in (O_2_)^−^ under drought, thereby significantly reducing lipid peroxidation and electron leakage compared to the control group.

ZnO NPs increased enzymatic and nonenzymatic antioxidant activities such as SOD, CAT, POX, glutathione reductase (GR), and APX recycling enzymes such as monodehydroascorbate reductase and dehydroascorbate reductase respectively. These enzymes aid in the reduction of oxidative damage caused by ROS production, thus protecting the seedlings from irrecoverable damage and death. ZnO NP treatment improves the uptake and utilization of Zn^2+^, which significantly increases the activity of SOD as it is Zn dependent. It has been suggested that this enhancement of antioxidant enzymes helps remove ROS generated due to drought stress, thus protecting plants from oxidative injury. ZnO NPs also activate the antioxidant defense system, protecting against oxidative injuries caused by drought stress (Ghani et al. 2022). TiO_2_ NPs and NO, supplemented with sodium nitroprusside, improved the gas exchange activity and photosynthetic parameters in wheat seedlings under drought stress. The upregulation of RuBisCo activase is involved in chlorophyll biosynthesis and carbon dioxide fixation. The treatment improved the uptake of Ca, Fe, Mg, and K in drought-stressed seedlings. These minerals are actively involved in photosynthetic activity and gas exchange. The wheat seedlings exhibited increased SOD, CAT, and APX activity. Although there was a significant increase in antioxidants, it was insufficient to protect the seedlings from ROS-caused oxidative injury due to drought stress. The addition of NO and TiO_2_ NPs increased the levels of antioxidants to the levels where they could combat oxidative injuries caused by ROS produced due to drought stress in wheat seedlings.

TiO_2_ NPs effectively regulate the antioxidant defense system, reducing lipid peroxidation by scavenging hydrogen peroxide (Faraji and Sepehri 2020). Cu NP-treated maize seedlings showed reduced chlorophyll content and increased carotenoid content. With the reduction in chlorophyll content, carotenoids in plants play the role of antioxidants by protecting chlorophyll against oxidative injuries caused by drought. Cu NPs reportedly reduce the ROS levels in maize seedlings under drought stress by increasing the activity of scavenging enzymes that aid in removing ROS from the plant system. Hence Cu NPs alleviate the oxidative stress caused by drought in maize seedlings by enhancing the photosynthetic parameters and enzymatic antioxidants, which strengthen and protect the seedlings from oxidative stress caused by drought (Nyuyen et al. 2022).

Plants produce osmoprotectants and osmolytes such as proline, glycine betaine, total soluble sugars, total proteins, and polyols under oxidative stress in response to drought. The increase in the production of such metabolites activates the stress tolerance mechanism in plants to overcome stressful environments. A sufficient build-up of osmolytes in plants can effectively maintain osmotic equilibrium and protect proteins, lipids, soluble sugar, and carbohydrates from oxidative damage, thus, preventing cellular damage as they are crucial cellular structures aiding in the fast recovery of stressed plants (El-Bassiouny et al. 2022). Recent reports from drought-stressed plants showed elevated levels of proline, glycine betaine, total proteins, and soluble sugars with NP treatment. In a study by Ghani et al. (2022), the levels of proline, glycine betaine, and amino acids were shown to be elevated with the treatment of zinc oxide nanoparticles on drought-stressed cucumber. In addition, ZnO NP treatment deterred soluble sugar reduction. Proline is an essential osmolyte that helps restore osmotic equilibrium, and ZnO reportedly increases proline biosynthesis by regulating gene expression in proline biosynthesis (Ghani et al. 2022).

### The effects of nanoparticles on phytohormones and gene expression under drought stress

Drought stress can activate numerous labyrinths of signaling pathways, which play a significant role in regulating the expression of drought-responsive and phytohormone-responsive genes. Metal/metal oxide NPs influence the expression of drought-inducible genes through two pathways, ABA-dependent and ABA-independent pathways (Linh et al. 2020), based on the type of plant and NPs used. Plant drought responses and defense mechanisms against tolerance are greatly influenced by phytohormones. ABA is the most critical phytohormone involved in mechanisms that operate in plants to overcome abiotic stress, including drought stress. ABA aids drought-stressed plants in overcoming drought and helps in attaining tolerance by regulating root development, leaf elongation, and the expansion of plants (Sallam et al. 2019). It also regulates stomatal conductance and thus controls transpiration rate and cellular water retention (Zahedi et al. 2021), while under severe drought, it also plays a major role as a signaling molecule in the biosynthesis of other phytohormones, including gibberellic acid, ethylene, and cytokinin (Sallam et al. 2019). García-Sánchez et al. (2015) studied the effects of TiO_2_ NPs on the transcriptome of *Arabidopsis thaliana* and found that drought stress could influence several NP-induced genes including transcription factors. MYBL2 regulates multiple signaling pathways, including jasmonic acid, SA, and ABA, pathways and COL5 which belongs to the CONSTANS family of flowering regulators.While the ABA-dependent signaling pathway helps ameliorate drought stress, COL5 regulates the flowering period of drought-stressed plants. Furthermore, this regulation might help the plant escape drought by shifting from a vegetative state to a flowering state.

The gene expression analysis reported by Linh et al. (2020) showed a higher expression of seven critical regulatory genes in NP-treated plants under drought conditions—*GmWRKY27, GmMYB118, GmMYB174, GmNAC11, GmRD20A, GmERD1,* and *GmDREB2*. Fe, Cu, Co and ZnO NP-treated soybean showed increased ABA content under drought stress. Treatment of plants under drought stress with Fe and Co NP treatment was found to upregulate the *GmWRKY27* gene expression, which is responsible for the biosynthesis of ABA. Higher expression of the *GmWRKY27* gene regulates ABA biosynthesis and signaling pathways involving ABA hormones. The *GmMYB118* gene was reported to improve drought tolerance by upregulating drought-related genes and subsequently reducing ROS levels, regulating osmolytes, and increasing flavonoid biosynthesis. In this study, NPs were reported to have penetrated cell membranes through membrane channels and formed complexes with calcium-binding proteins which were suggested to promote the expression of genes that help to overcome drought. This study indicates that NP treatment of plants under drought stress induced tolerance by regulating the gene expression of drought-related genes and promoting the biosynthesis of ABA (Linh et al. 2020). Transcriptomic studies have revealed that CNTs can upregulate stress-related genes in both monocotyledonous and dicotyledonous plants (Rezaei Cherati et al. 2021).

### The effects of nanoparticles on the life cycle, yield, and nutritional quality of crops under drought stress

Unfavourable environmental conditions such as drought stress can significantly impact the physiology of the crop, yield, productivity, and quality. Drought-induced yield loss has adverse effects that extend beyond individual farming households to entire farming communities and, in some cases, countries as a whole (Ahmed et al. 2021a). Soybean seedlings subjected to drought stress exhibited a significant decrease in growth indices during the vegetative and reproductive phases. However, seedlings treated with micnobits (mixture of micronutrients such as ZnO, CuO, and B_2_O_3_ NPs of different sizes) enhanced the growth indices similar to plants grown in non-drought conditions. The leaf area, number, and fresh shoot weight were enhanced during vegetative maturation. Similarly in vegetative growth, drought results in a substantial negative impact on soybean reproductive parameters such as pod fresh weight, number, fresh grain weight, grain count, and grain dry weight in comparison to the plants that received an optimal supply of water. Under drought stress, micnobit treatments enhanced grain fresh weight, pod fresh weight, and pod number. The foliar application of micnobits increased the yield and helped the soybean plant recover from drought stress efficiently. The study also reported a significant spike in the translocation of nutrients, such as N, and K, in the grain (Dimkpa et al. 2017). The wheat seedlings treated with Cu and Ag NPs using hydroponics showed enhanced morphological and yield indices compared to controls. Plants treated with Cu NPs (3 mg/L) and Ag NPs (30 mg/L) showed higher spike length, spikelet density per spike, grain weight, and number in stressed seedlings compared to other treatments and control groups (Ahmed et al. 2021a).

Linseed treated with TiO_2_ NPs increased the seed oil and protein content and thereby yield quality under water-stressed conditions. TiO_2_ NPs at 100 mg/L enhanced the seed oil content to 37 and 35% and seed protein content to 19 and 22% in well-watered and stressed conditions, respectively (Aghdam et al. 2016). Treatment with NPs can aid plants in coping with drought stress in both vegetative and reproductive stages of the plants. NP treatment can be promising in improving yield quantity and quality under unfavorable conditions. Further investigation to confirm and understand the role of nanoparticles in improving plant life throughout its cycle under drought stress needs urgent attention. In addition, studies on the accumulation of NPs in grains and their effects on human and livestock health upon consumption need to be studied.

### The effects of NPs on secondary metabolites under drought stress

Secondary metabolites are known as markers of stressful conditions in plants. Stressed plants produce high amounts of ROS, which cause oxidative stress, and these secondary metabolites act as a plant defense system against stress (Yadav et al. 2021). Secondary metabolites such as phenolics, flavonoids, and alkaloids have therapeutic applications that pique research interest (Ali et al. 2021). However, the mechanism by which NPs are involved in the biosynthesis of secondary metabolites is still unknown. Water-stressed sorghum seedlings grown in Si NP-treated soil showed enhancement in total phenolic compounds and a significant increase in flavonoid content. Ferulic acid, a phenolic compound, was found to be significantly higher in mild and moderately stressed plants; however, it was reduced in severely stressed seedlings. Vanillic acid was significantly higher in severely stressed seedlings than in mildly and moderately stressed seedlings. The biosynthesis of phenolic compounds could be responsible for the fortification of the cell wall by Si NPs (Ghorbanpour et al. 2020). Foliar treatment of chitosan NPs in periwinkle seedlings showed a significant increase in alkaloid contents under stressed conditions. The accumulation of alkaloids was higher in root samples than in shoot samples. Increased alkaloid content improved plant growth by enhancing water uptake through osmotic adjustment. The study also suggests that the transportation and storage of photosynthates was attributed to the high alkaloid content. Chitosan NPs reportedly upregulated the genes coding for strictosidine synthase (STR), deacetylvindoline-4-*O*-acetyltransferase 15(DAT), peroxidase 1 (PRX1) and geissoschizine synthase (GS), which are involved in the biosynthesis of terpenoid indole alkaloids (TIAs).

The exact mechanism behind interspecies variation in nanoparticle uptake and drought amelioration is still under investigation. Plant-nanoparticle interactions vary from species to species and is primarily influenced by the type of nanoparticle. Anatomical variations in plants such as pore (size, density, and distribution), stomata (size, density, and distribution), epidermal thickness, and composition can affect NP-plant interactions (Rasheed et al. 2022). The effects of nanoparticles in alleviating drought stress in plants must be investigated further to understand the underlying mechanisms. NPs seemingly enhance the uptake and utilization of nutrients; however, the involvement of NPs in nutrient pathways and ionic channels must be explored. Although, it is well reported that NPs improve photosynthetic parameters and increase antioxidant activities, the mechanism by which NPs influence the intercellular pathways of photosynthetic reactions and activation of antioxidants largely remain unexplored. Moreover, an omics-based approach could reveal the effects of NPs on plants under drought stress and their mechanism of stress tolerance. The use of NPs in the alleviation of drought stress in crop plants could be an innovative approach in the field of agriculture. However, overusing NPs can lead to high dosage accumulation in food grains, which can enter the food chain causing high risk. Care should be taken to prevent toxic side effects of NPs before marketing nano-Agri products.

## Conclusion and future prospects

Nanoparticle treatment has been reported to alleviate drought stress in host plants via enhanced physiological parameters from the regulation of water relations, stomatal conductance, photosynthetic activity, and improved uptake of micro- and macro-nutrients to NP-based fertilizers. In this review, we have comprehensively analyzed the mechanisms of uptake and beneficial effects of nanoparticles under drought stress in monocotyledonous and dicotyledonous plants. Several reports have highlighted that the germination, morphological, and physio-biochemical parameters of drought-stressed crop plants could be significantly enhanced using nanoparticle treatment. In addition, due to nanoparticle exposure, the signaling pathways related to the biosynthesis of phytohormones and osmoprotectants were shown to be upregulated during drought stress. However, the precise mechanisms underlying nanoparticle transportation and its impact at the molecular level in drought-stressed crops remain unknown. In this review, we have compiled recent advancements in NPs that provide a better understanding of the mechanisms of uptake, plant-nanoparticle interactions, compartmentalization, and their beneficial role in the host plants.

## Abbreviations

BTU: British Thermal Units
GDP: Gross Domestic Product
CAT: Catalase
SOD: Superoxide Dismutase
APX: Ascorbate
POD: Peroxidase
GSH: Glutathione
ROS: Reactive Oxygen Species
NMs: Nanomaterials
NPs/NP: Nanoparticles/nanoparticle
PVP: Polyvinylpyrrolidone
Nramps: Natural Resistance-Associated Macrophage Proteins
IRT1: Iron Regulated Transporter 1
COPT1: Copper Influx Transporter 1
FT-IR: Fourier Transform Infrared
MWNTs: Multiwalled Carbon Nanotubes
SWNTs: Single-Walled Carbon Nanotubes
SEL: Size Exclusion Limit
PEG: Polyethylene Glycol
TEM: Transmission electron microscope
MDA: Malondialdehyde
ABA: Abscisic acid
SA: Salicylic acid
STR: Strictosidine synthase
DAT: Deacetylvindoline-4-*O*-acetyltransferase 15
PRX1: Peroxidase 1
GS: Geissoschizine synthase
TIA: Terpenoid indole alkaloids
